# Investigating mental representations of psychoactive substance use and other potentially addictive behaviors using a data driven network-based clustering method

**DOI:** 10.1371/journal.pone.0287564

**Published:** 2023-10-19

**Authors:** Domonkos File, Bálint File, Beáta Bőthe, Mark D. Griffiths, Zsolt Demetrovics

**Affiliations:** 1 Institute of Psychology, ELTE Eötvös Loránd University, Budapest, Hungary; 2 Wigner Research Centre for Physics, Budapest, Hungary; 3 Département de Psychologie, Université de Montréal, Montreal, Canada; 4 Psychology Department, International Gaming Research Unit, Nottingham Trent University, Nottingham, United Kingdom; 5 Centre of Excellence in Responsible Gaming, University of Gibraltar, Gibraltar, Gibraltar; Ladoke Akintola University of Technology Teaching Hospital: LAUTECH Teaching Hospital, NIGERIA

## Abstract

**Background and aims:**

The aim of the present study was to examine the mental representations of the use of different substances and other potentially addictive behaviors in order to explore meaningful similarities and differences that may contribute to a better understanding of behavioral addictions’ representations and diagnostic criteria.

**Methods:**

The authors mapped the mental and emotional representations of 661 participants (70.5% women; *M*_*age*_ = 35.2 years, SD = 11.7) to the concept "your most disturbing excessive activity" using free-word associations combined with a network-based clustering method.

**Results:**

The network analyses identified four distinct mental representations, three implicating dominantly negative (Guilt/Shame/Relief, Addiction/Health, and Procrastination/Boredom) and one dominantly positive emotion (Stress/Relaxation). The distribution of Addiction/Health and Procrastination/Boredom representations were different across substance use and problem behaviors, indicating meaningful differences in the underlying cognitive evaluation processes. The Addiction/Health representation was more frequent for substances, while for other addictive behaviors, the Procrastination/Boredom representation was more frequent, and its frequency increased with the self-reported intensity of the behavior. Guilt/Shame/Relief was equally common for both substances and behaviors, but importantly, for substances its’ likelihood increased with the intensity of use.

**Conclusion:**

The common part of representations for substance use and other potentially addictive behaviors supports the scientific viewpoint, that real addictions can exist even in the absence of psychoactive drugs. Based on the results, a novel proposition is posited, that a more appropriate indicator of tolerance for problem behaviors might be the perceived amount of time wasted on the activity rather than the actual time spent.

## Introduction

Growing evidence suggests that behavioral addictions (e.g., addictions to gambling, gaming, sex, etc.) share significant similarities with substance use addictions in many domains, such as the etiology, tolerance, comorbidity, and response to treatment [1–5] Besides the similarities, consensus about the applicability of some factors has not been reached such as physical signs of addiction [6] and tolerance [7,8].

A major critique against behavioral addiction research is that the diagnostic components are inherited from substance addiction practice, often leading to the negligence of the heterogeneity of these disorders [9]. For example, tolerance, one of the key features of addiction, is a highly debated criterion [10,11]. As Billeux et al. (2015b) illustrate, no-one would argue if someone started to play the guitar and spends more and more time with it, developing tolerance towards the behavior and/or “music addiction”. Similarly, mood modification has low specificity in case of internet gaming disorder [12]and problematic pornography use [13] (i.e., a significant proportion of non-addicted users also engage in the activity to modify their mood). As Griffiths [10] points out, evaluating the balance of positive and negative consequences is necessary to distinguish between excessive enthusiasms and addictions. If positive consequences outweigh negatives, the behavior in question cannot be defined as an addiction. To define such a balance, a multivariate function is required, for which the exploration of possible dimensions with high explanatory power can increase the accuracy of diagnosis. Also, considering the lack of consensus concerning the common diagnostic components, and the often not straightforward interpretability in case of behavioral addictions [9,14,15], introducing novel empirical approaches to further explore differences and similarities might be beneficial for either scientific purposes or clinical use in the long-term.

A potentially useful approach to acquire the adequate frames is to map the structure and content of mental representations (MRs) towards their own addiction from individuals with behavioral or substance addiction. A widely used tool to examine MRs is the free word association (FWA) task, through assessing the first ideas elicited by a cue (stable implicit attitudes by verbal associations). FWAs are considered as a technique for overcoming the limitations of predetermined frames by exploring representations beyond the researcher’s preconceptions. Similar to open-ended questions, the FWA task allows participants to describe their beliefs and experiences in their own words [16]. Although classical open-ended questions provide more detailed information than FWAs, the higher complexity of texts require more advanced natural language techniques, which are still not well-established measures [17,18]. Some researchers even argue that these answers assess the ability of the participant to articulate the sentences and not their opinions [19]. It has also been suggested that FWAs related to engagement in cue-relevant behaviors [20] because past behaviors generate associations in memory that bias future behavior. Given the potential of the FWA task to provide unrestricted access to MRs, their exploration may help to understand the mechanisms underlying behavioral and substance addictions.

Addiction research using FWA techniques began with the work of [21] Szalay et al. (1992), who focused on the representational differences of individuals with and without substance use disorder, regarding both substance-specific topics (“drug” [22]) and general topics (e.g., “me”, “family”, “father” [21]). They reported meaningful differences in both perceptual and motivational dispositions between pretreatment and rehabilitated clients, with possible consequences on treatment evaluation [21,22]. Stacy et al. [23] presented a series of alcohol-related and neutral short phrases to college students who were asked to generate the first behavior that came to mind. Strong associations were found between the generation of alcohol responses and alcohol consumption, indicating the importance of memory processes in alcohol use in a non-clinical sample [23]. Reich, Goldman & Noll [24] investigated alcohol-related representations on a large college sample. They found that heaviest alcohol drinkers had more positive and arousing responses than did lighter alcohol drinkers, who had more negative and sedating responses. The results were also interpreted as evidence of the influence of memory processes on the behavioral response to alcohol consumption [24]. In another study, cigarette smoking-related positive and negative information was assessed with an FWA test across smokers and non-smokers [25]. They have found, that although smokers generated more positive smoking-related associations than non-smokers, in total, both groups produced more negative associations. Cigarette smokers in the early time interval generated proportionally more positive associations, suggesting that positive smoking-related memories were easier to activate, even though they also have more negative associations available [25].

Although FWA techniques are capable of identifying meaningful MRs explaining addiction, research using this technique has remained scarce in this field. In addition, these techniques have used human raters to identify relevant FWA groups. If FWAs are considered as a technique for overcoming the limitations of predetermined questions by exploring representations beyond the researcher’s preconceptions, then the clustering of FWAs by the researcher may also hinder this goal. To overcome this limitation, there are a few clustering solutions tried by previous studies, such as factor analytic methods [26] and hierarchical agglomerative clustering [27]. The current study implemented a modular analysis of the co-occurrence network of free word associations (FWAs) [28]. We applied this approach, as our previous methodological research has demonstrated the capability of modular analysis in establishing a reproducible result [28] and connecting mental representations (MRs) to cue-specific behaviours and attitudes of participants [28,29].

With an exploratory intent, the present study investigated possible differences between the (i) MRs of potentially addictive substances and behaviors, (ii) emotions linked to the MRs of potentially addictive substances and behaviors, and (iii) structure of MRs as a function of subjective severity of the substance use and other potentially addictive behaviors.

## Materials and methods

### Participants

The samples were collected via online questionnaires, and survey completion took

approximately 20 minutes. The sample used in this study may not be representative of the Hungarian population, as it was obtained through a popular Hungarian news portal and public, topic-irrelevant Facebook pages. The study was advertised as a research project concerning the psychological factors of excessive activities, and data were collected from June to September in 2020. Informed consent was obtained from participants before data collection, and participants were ensured of their anonymity. The present study was conducted adhering to the Declaration of Helsinki and was approved by the institutional ethical review board of the Eötvös Loránd University (2020/258). No personal information that allowed personal identification was asked, and a secure online platform (Qualtrics Research Suite; Qualtrics, Provo, UT) was used for data collection. Required sample size was determined based on a previous study using the same methodology [30].

The inclusion criteria were (i) providing informed consent and (ii) being aged 18 years or older and (iii). Overall, 661 participants (466 women, 70.5%) aged between 18 and 74 years (*M*_*ag*e_ = 35.2 years, SD = 11.73) completed the survey. Of these, 2.3% had maximum primary education, 5.9% reported having a vocational degree, a further 15.3% had high-school degree, and 76.4% college or university degree. Regarding relationship status, 185 were single (27.9%), 458 were in any kind of romantic relationship (i.e., being in a romantic relationship or married) (69.3%), 17 chose the “other” option (2.6%), and one participant did not provide a response to this question Table 1 presents the demographic characteristics of the sample.

**Table 1:** Overview of the demographic characteristics of the sample.

	%	Mean	SD	Range
Age		35.2	11.7	18–74
Gender (female)	70.5			
**Completed education**				
Primary education	2.3			
Vocational degree	5.9			
High-school degree	15.3			
College or university degree	76.4			
**Relationship Status**				
Single	27.9%			
In romantic relationship	69.3			
Other	2.3			

### Procedure and measures

Participants were asked to write five words or expressions that comes into mind regarding the sentence “Imagine you are doing the excessive activity that bothers you the most”. We have deliberately chosen to use “excessive activity” instead of using the word ‘addiction’ as a call word, as (1) abstract, often used expressions easily trigger common knowledge rather than subjectively shaped MRs, (2) the word ‘addiction’ is highly stigmatized [31] and might leads to resistance, and (3) different interpretations of the word would make it difficult to interpret the results. After providing all five associations, participants got back their associations one-by-one and were asked to select two emotional labels from the list of 20 Positive and Negative Affect Schedule (PANAS) labels [32] to each of the associations. Using emotions to enhance the analysis of free-word associations has proven to be of benefit in recent studies [28].

Participants were then asked to indicate the substance/behavior they thought of from a list of four psychoactive substances and ten potentially addictive behaviors (see Table 2). If the substance/behavior in question was not on the list, an "other" option could be chosen. After this, a subjective evaluation of the problem severity was indicated using one item (*“I did it too much in the past 12 months”*) taken from the Screener for Substance and Behavioral Addictions (SSBA, [33]) comprising four response options: ‘Totally disagree’, ‘Partly disagree’, ‘Partly agree’, ‘Totally agree’.

**Table 2:** The distribution and demographic characteristics of the investigated substance uses and behaviors.

	N of responses	Severity of problematic activity	Gender of respondents (% of females)	Age of respondents
Alcohol use	66	3.5 (0.75)	69.7%	34.16 (8.62)
Tobacco use	87	3.72 (0.58)	67.8%	37.24 (12.45)
Cannabis use	22	3.68 (0.57)	45.5%	31.22 (7.82)
Other substance use	11	3 (1.34)	63.6%	28.27 (4.94)
Social media use	85	3.6 (0.71)	87.0%	30.33 (10.23)
Internet use	65	3.57 (0.66)	76.9%	33.4 (11.27)
TV series watching	25	3.56 (0.65)	84.0%	33.16 (12.46)
Eating	73	3.60 (0.55)	93.15%	36.33(10.81)
Work	39	3.79 (0.57)	61.5%	39.76 (12.34)
Pornography use	33	3.48(0.83)	12.1%	36.53 (12.47)
Shopping	19	3.26(0.93)	89.5%	38.42 (11.64)
Gaming	38	3.39 (0.75)	44.7%	37.61 (11.48)
Sex	17	3.06 (1.08)	47.1%	42.58 (11.62)
Gambling	5	2.8 (1.09)	20.0%	45.2 (15.99)
Other behaviors	76	-	78.9%	36.71 (13.85)

### Analyses

Free-word associations were (i) first spellchecked (transformed to lower case, removed accents, and manually corrected), (ii) lemmatized and (iii) translated to English. Associations were merged if their English translation was identical, or was a close synonym. While assessing the substance/behavior the participants thought of, 76 (out of 661) participants indicated ‘other’ from the list of choices (see Table 2). As it is not known whether their associations concerned a substance or a behavior, these participants were excluded from further analysis. FWAs mentioned by less than 10 participants were excluded from the analysis as such terms can refer to unstable or idiosyncratic parts of the representations [34,35] and a breakpoint at a frequency of 10 was observable (see figure at Appendix), indicating that excluding associations mentioned less than 10 times still retained 65% of the total FWAs in the analysis. Participants whose associations’ frequency for each category was lower than 10 were unable to be classified (n = 40) and were subsequently excluded from subsequent analyses. This resulted in a final study cohort consisting of 546 participants. PANAS labels were considered relevant in the context of the study that were provided by at least 50% of participants overall. The decision to set this threshold was arbitrary, serving the purpose of maintaining stability in emotional labels and directing the study towards emotional states that, due to their frequent occurrence, were more likely to have a general consensus among the respondents. S2 Appendix contains the results pertaining to the labels that were not considered due to the implementation of this approach.

The present study applied multiple FWAs (five FWAs/individuals) with a data-driven method (i.e., associations were connected base on their statistical co-occurrences [CoOp] method; see [28]]), in which FWA groups are derived from patterns of individual representations. More specifically, networks were constructed from the statistical co-occurrence of the FWAs and distinct MRs were identified as densely connected FWAs. It has been demonstrated in recent studies that MRs identified by FWAs from the aforementioned network procedure can create a stable structure and can be linked to the cue-specific behavior and attitudes of the participants [28,29].

In this analysis, major MRs of addiction were extracted from the network analysis of FWAs. In this network, each unique association was defined as a node and co-occurrence relationships between the associations as edges. Statistical co-occurrence between every pair of associations was defined as the loglikelihood ratio between the maximum likelihood of the observed co-occurrence and the likelihood assuming statistical independence. Associations were *attractive* (i.e., attained a positively weighted edge) if they were mentioned more frequently together than by chance or *repulsive* (i.e., attained a negatively weighted edge) if they were mentioned less frequently together than by chance. Higher deviation from chance indicated higher negative or positive weight. Densely connected subnetworks were identified as modules. These modules reflecting different MRs regarding *“most disturbing activity”*. Modules were defined by consensus clustering from the maximal modularity partitions of the network identified by the Louvain algorithm [36,37]. Due to the negative edges in the network, an extended formula of modularity was applied:

$$Q=\frac{1}{v^{+}+v^{-}}\sum_{ij}[\left(w_{ij}^{+}-e_{ij}^{+})-(w_{ij}^{-}-e_{ij}^{-}\right)]\delta_{M_{i}M_{j}}$$

(i) Q denotes the modularity value of a given partition of a network, (ii) *v*^+^/*v*^−^ denotes the total positive/negative weights of the network, (iii) $w_{ij}^{+}/w_{ij}^{-}$ denotes the positive/negative weights between node *I* and *j*, (iv) $e_{ij}^{+}/e_{ij}^{-}$ denotes the chance-expected positive/negative connections between node *I*, and (v) *j*, $\partial_{M_{i}M_{j}}$ is an indicator function that is set to 1 if node i and j belong to the same module.

The emotional features of the association modules were described by the emotional labels of the associations. The relative percentage distribution of the emotions in each module was calculated. To characterize the emerging modules with substances or behaviors, each participant was linked to the module from which most of their associations came from.

Chi-square tests of independence were performed to examine the relationship between misuse type (substance or behavioral) and MRs. Also, chi-square tests of independence were performed to examine the relationship between problem intensity (low, high) and MRs for substance uses and behaviors separately. Problem intensity was defined on the basis of the SSBA item (*“I did it too much in the past 12 months”*). Participants who indicated ‘Totally disagree’, or ‘Partly disagree’ or ‘Partly agree’ were classified into the low-intensity group, whereas participants who indicated ‘Totally agree’ were classified into the high-intensity group. Kruskal-Wallis H tests were performed to explore possible differences (i) between emotions of psychoactive substances and potentially addictive behaviors, and (ii) between emotions related to the four emerging representational modules.

The network construction was performed with MATLAB version R2019b [38] (The MathWorks Inc, Natick, MA). The applied network measures are available at https://sites.google.com/site/bctnet/ [39]. Visualization of the network was done by Gephi 0.9.2 [40].

## Results

The total number of unique associations was 1374. After the spellcheck, lemmatization, and merging, the number of associations decreased to 254, of which 77 were non-idiosyncratic and represented the basis of the network analysis. It is important to stress that the 77 unique association covered 65% of the total number of produced FWA answers (for further details see S1 Fig in S2 Appendix).

For the item *“I did it too much in the past 12 months*”, 3% indicated ‘Totally disagree’, 3% ‘Partly disagree’, 25.8% ‘Partly agree’, and 68.2% ‘Totally agree’.

**Fig 1:**
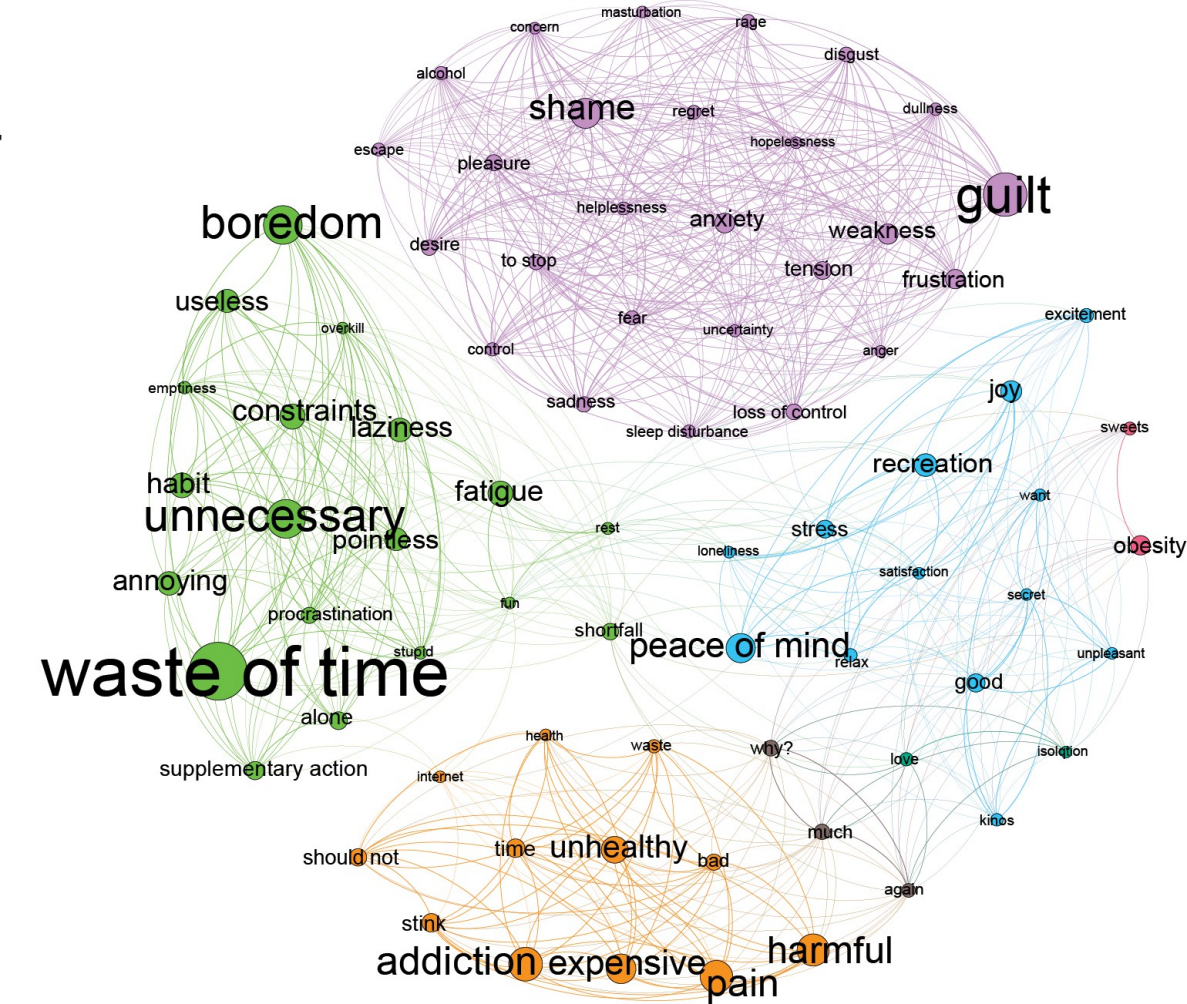
Mental representations of the CoOp networks. Each MR is visualized with different colors (purple:Guilt/Shame/Relief; blue: Stress/Relaxation; orange: Addiction/Health, green: Procrastination/Boredom). The sizes of a node and its label are proportional to the frequency of the given association. An edge means that two associations fall into a common MR in the consensus-partitioning procedure at least 40%.

The network analyses identified four distinct MRs which were subsequently labeled as Guilt/Shame/Relief, Addiction/Health, Stress/Relaxation, and Procrastination/Boredom(see Fig 1).

**Fig 2:**
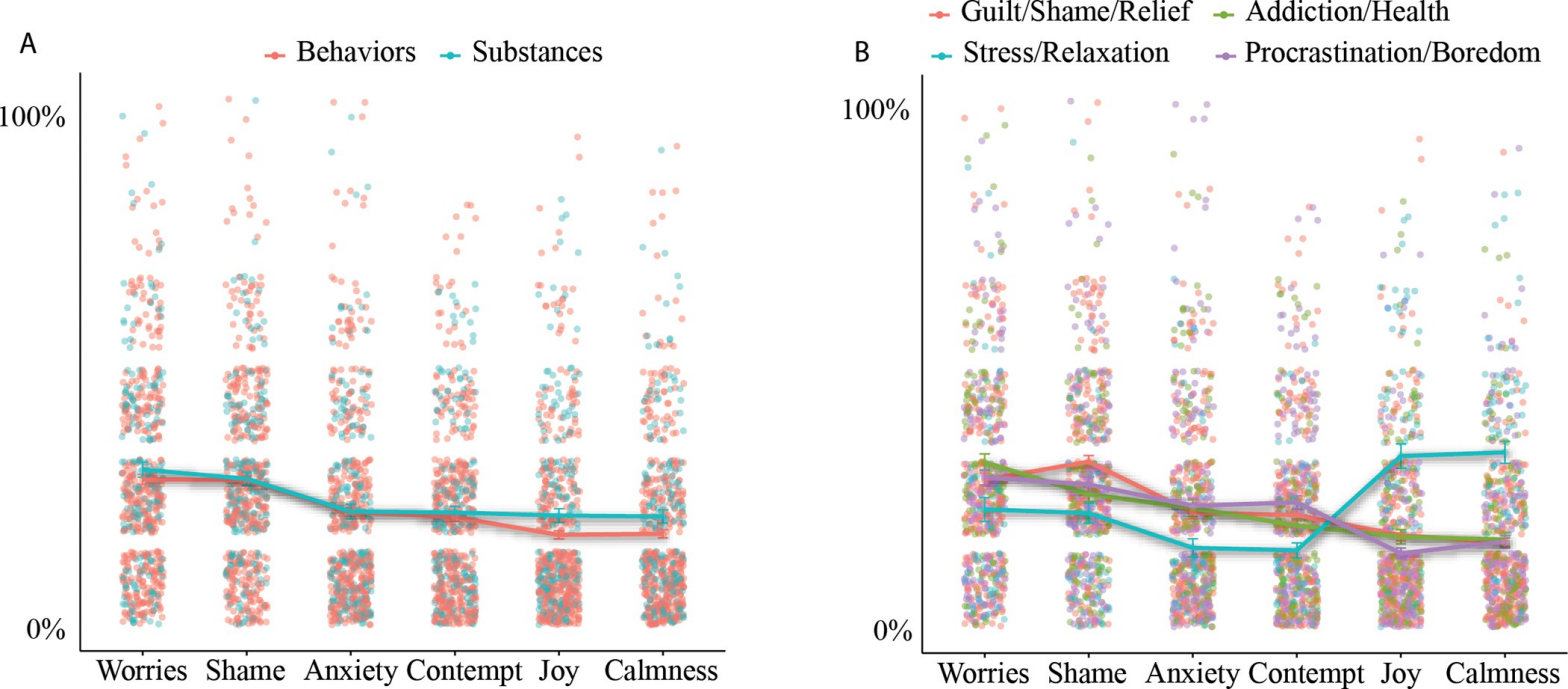
Graphical representation of the differences in emotions between (A) psychoactive substance uses and potentially addictive behaviors and (B) between MRs.

Six dominant emotions were covered by the 51% of the selected PANAS labels: worries, shame, anxiety, contempt, joy, and calmness. The distribution of these emotions was different across MRs (‘worries’ [H = 14.6, df = 3, *p* = 0.002]; ‘shame’ [H = 23.8, df = 3, *p*<0.001]; ‘anxiety’ [H = 13.7, df = 3, *p* = 0.003], ‘contempt’ [H = 19.9, df = 3, *p*<0.001], ‘joy’ [H = 65.1, df = 3, *p*<0.001], ‘calmness’ [H = 76, df = 3, *p*<0.001]). The outcomes of pairwise comparisons using Dunn’s tests are reported in Table 3 and Fig 2.

**Table 3:** Pairwise comparison of emotions across mental representations.

	Worries	Shame	Anxiety	Contempt	Joy	Calmness
Guilt/Shame/Relief-Stress/Relaxation	0.02	<0.001	0.003	0.003	<0.001	<0.001
Addiction/Health-Stress/Relaxation	<0.001	-	0.002	0.02	<0.001	<0.001
Procrastination/Boredom—Stress/Relaxation	0.02	0.04	0.004	<0.001	<0.001	<0.001
Addiction/Healt-Procrastination/Boredom	-	-	-	0.04	-	-
Guilt/Shame/Relief-Procrastination/Boredom	-	0.01	-	-	-	-

Numbers indicate p-values.

**Fig 3:**
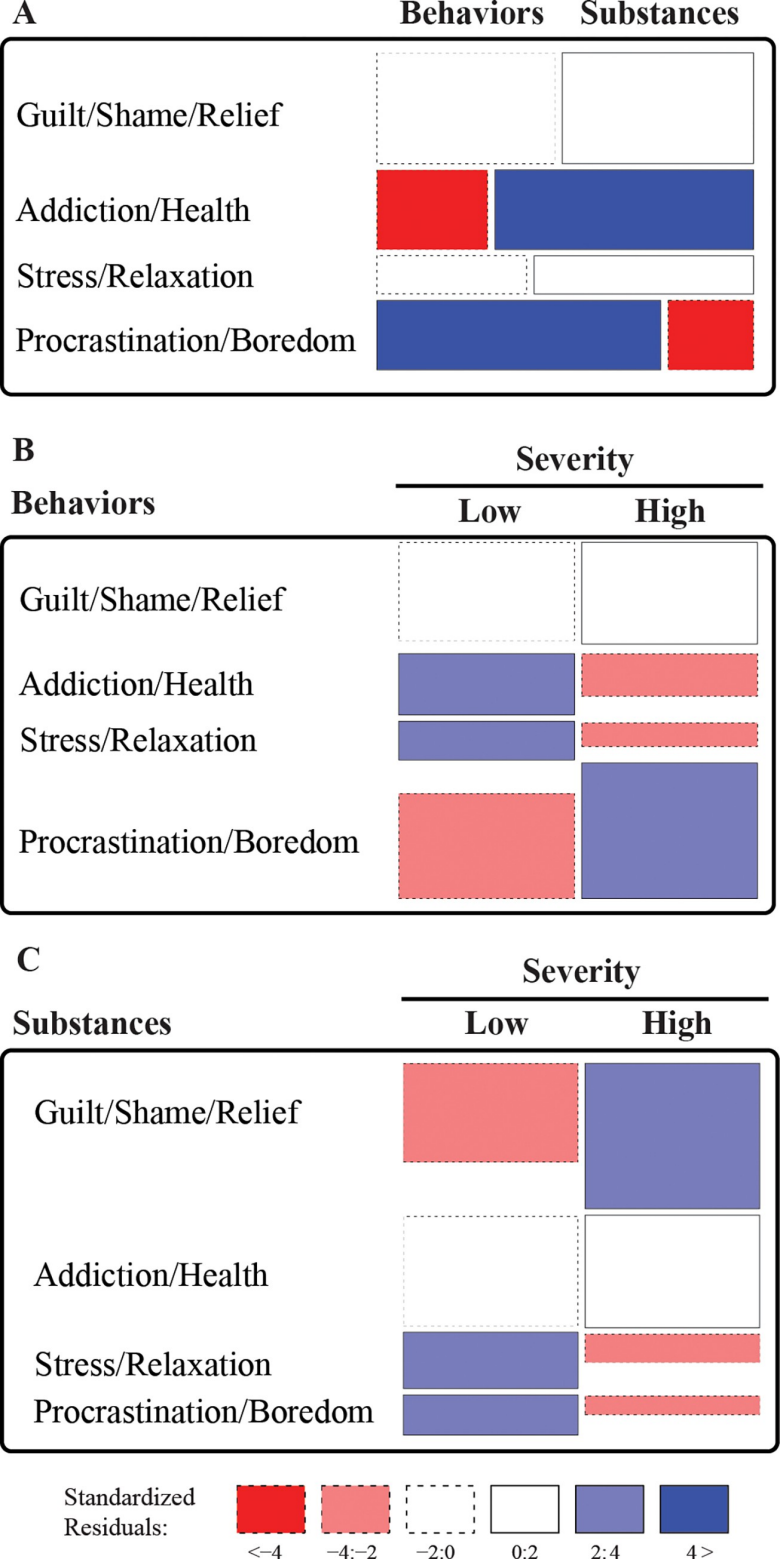
Graphical representation of differences in MRs between (A) psychoactive substance uses and potentially addictive behaviors, (B) low-intensity and high-intensity potentially addictive behaviors, and (C) low-intensity and high-intensity psychoactive substance uses. Colors indicate how far the observed frequencies deviate from what would be expected under the assumption of independence between the variables.

The relationship between problem type and the related MRs was significant (χ^2^[df = 3, *N =* 546] = 103.3, *p* < .0.0001). Associations from the Addiction/Health representation were more likely in case of psychoactive substances, while associations from the Procrastination/Boredomrepresentation were more likely for potentially addictive behaviors (Fig 3A). The relationship between intensity and representations were significant for both psychoactive substances (χ^2^[df = 3, *N =* 180] = 85.0, *p* < .0.0001 and potentially addictive behaviors (χ^2^[df = 3, *N =* 366] = 36.0, *p*<0.0001 (Fig 3B and 3C). In case of high intensity substance use, associations were more frequent in the Guilt/Shame/Relief representation, while in case of low intensity substance use associations, the Stress/Relaxation and Procrastination/Boredom representations were more frequent. In case of high intensity potentially addictive behaviors, associations were more frequent in the Procrastination/Boredom representation, while in case of low intensity problem behaviors associations, the Relaxation and Addiction/Health representations were more frequent. There was no difference regarding emotions and positive and negative affects between low-intensity and high-intensity psychoactive substance uses and potentially addictive behaviors.

Comparing emotions between psychoactive substance uses and potentially addictive behaviors across the four modules using the Kruskal-Wallis chi-squared test only identified significant differences between the positive emotions of ‘joy’ (H = 7.3, df = 3, *p* = 0.006) and ‘calmness’ (H = 6.6, df = 3, *p*<0.01)–see Fig 2A. That is, ‘joy’ and ‘calmness’ were indicated more often for the associations of psychoactive substance uses.

## Discussion

Previously, no explorative data-driven studies have ever described the differences between the mental representations (MRs) of psychoactive substance use behaviors and other potentially addictive behaviors, despite preliminary evidence suggesting FWA’s applicability and usefulness in understanding different potentially addictive behaviors (e.g., [21–23]). Therefore, the aim of the present study was to fill this gap by mapping mental and emotional representations of the concept of individuals’ *“most disturbing excessive activity"* using free-word associations combined with a data-driven clustering method [28] and linking these representations to specific measures of substance uses and potentially addictive behaviors. The analysis showed that the perception of *“disturbing excessive activity”* formed four distinct MRs, three with dominantly negative emotions (Guilt/Shame/Relief, Addiction/Health and Procrastination/Boredom) and one with dominantly positive emotions (Stress/Relaxation) (see Fig 2B).

*Guilt/Shame/Relief* (35%): The most frequent MR included associations of diffuse concepts, such as “guilt”, “shame”, “weakness”, “anxiety” and “frustration” which suggests generalized representations not limited to the psychoactive substance or potentially addictive behavior. Guilt has been described previously as a primary affective response in case of alcohol and other substance use disorders [41], tobacco use [42], problematic social networking site use [43] and compulsive sexual behavior and problematic pornography use [44–48]. Both “shame” and “guilt” were frequent associations, suggesting that the suspected view upon the self of others and self-reviewing and self-judgment can be crucial mechanisms [49], either restricted to the addiction–guilt -, or generalized to self-identity–shame–[41], but not differentiated strictly among individuals’ MRs.

*Addiction/Health* (21%): Since frequent associations of this representation were “addiction”, “pain”, “harmful”, and “unhealthy” (i.e., concepts belonging to health), it can be hypothesized that the concept of addiction and health risks are closely connected. The dominating emotion of this module was worries, presumably about health-related issues, in line with the extensive literature on health-related harms and substance addiction (e.g., [50]).

*Procrastination/Boredom* (29%): Frequent associations within this representation were “waste of time”, “unnecessary”, “boredom”, “constraints” and “fatigue”, all of which reflects passive acceptance rather than an active involvement. Previous research has described a possible causal link between leisure-boredom and substance abuse [51] and different behavioral addictions (internet: [52,53], sex: [54], pornography: [55,56], and smartphone use and compulsive shopping: [57]).

*Stress/Relaxation* (15%): Frequent associations were dominantly positive within this representation; “calm”, “recreation”, “joy” and “good” with only one negative (i.e., “stress”). The dominating emotions were also positive (e.g., joy, relaxation) but negative emotions were also present (e.g., worries, anxiety), although with lower likelihood than in the other three representations (see Fig 2A and Table 3). Relaxation, as a motivational factor has been described in case of many potentially addictive behaviors or psychoactive substance use, such as in case of alcohol use [58], tobacco use [59], cannabis use [60], overeating [61], gaming [62,63], hedonic shopping [64], problematic internet use [65], pornography use [55,56], and social media use [66].

Comparing the MRs with psychoactive substance uses and potentially addictive behaviors, a number of important differences were observed. Importantly, the distribution of Addiction/Health and Procrastination/Boredom MRs were different across substance uses and problem behaviors (see Fig 3A), indicating meaningful differences in the underlying cognitive evaluation processes. In case of behavioral addictions, one crucial measure of problem severity is the time spent on the activity [67]. Since there is no substance involved, tolerance is also reflected in the amount of time engaged with the activity, which can lead to the significant overpathologization of everyday life activities [9,68]. Based on the present study’s results, it is proposed that it is not the time spent on the behavior *per se* that is problematic, but what the individual judges to be the time wasted from it, which might be a better indicator of the severity of behavior-related problems. This also supports Griffiths’ [12] contention that time (in and of itself) should not be used as a criterion to assess behavioral addictions and that it is how the time conflicts with other important activities that is key. To illustrate with the aforementioned guitar playing example [9], assessing solely the time allocated to guitar playing is not sufficient to determine tolerance/addiction. However, if completed with the assessment of perceived wasted time, high scores will possibly reflect the level of tolerance. This reasoning is supported by the fact that Time-wasting MRs were more frequent among individuals reporting high severity involvement in a behavior compared to low severity. While time wasting activities are not explicitly included in the diagnostic criteria of the ICD-11 [69], excessive time spent on a behavior that interferes with other important areas of life may be related it. For Gaming Disorder, the ICD-11 criteria state that the gaming behavior "takes precedence over other life interests and daily activities" and that the individual continues to engage in gaming despite "the occurrence of negative consequences", which might also lead to the percept of wasted time. For Gambling Disorder "gambling is often accompanied by a preoccupation with the activity" that can interfere with other important areas of life. Similarly, while the I-PACE model doesn’t explicitly mention time wasting as a criterion, it does recognize that excessive engagement in certain activities (such as gaming, internet use, or gambling) can lead to negative consequences and interfere with daily functioning [70]. Thus, time wasting could be considered as a consequence or outcome of addictive behaviors rather than a standalone criterion within both the ICD-11 and I-PACE approach.

Participants with potentially addictive substance use habits mentioned associations from the Addiction/Health MR more frequently, indicating that individuals tend to classify their “disturbing excessive activity” as addiction if a substance is involved. This is in line with previous findings that non-professional participants perceived activities involving drug use (e.g. alcohol, smoking) to be more addictive than those that do not (e.g. exercise, watching TV) [71]. This might reflect the long-held scientific viewpoint–although this is now changing with the inclusion of behavioral addictions into the *Diagnostic and Statistical Manual of Mental Disorders* [72] and *International Classification of Diseases* [69]. This is also in line with the rich scientific literature on the health consequences of substance use addictions (e.g., [50,73]), and that the awareness of health hazards related to addictive substances is relatively high (e.g., [74,75]). Another factor that may contribute to the observed difference is that behavioral addictions arguably have a less direct negative or immediate impact on health (e.g., excessive internet use does not directly affect health, but can contribute to a decrease in physical activity, which can lead to a negative assessment of physical health [76]).

Guilt/Shame/Relief was an equally important MR for both substance misuse and potentially addictive behaviors, suggesting that self-criticism and evaluation [49] are determinative processes irrespective of the focus of the substance or behavior. However, importantly, the dynamics of the underlying cognitive-psychological processes might be different. For individuals with high-intensity substance use, Guilt/Shame/Relief was more frequent compared to low-intensity substance use, and Stress/Relaxation and Procrastination/Boredom were more frequent for low-intensity substance use. In other words, while in case of low-intensity use, all four representations were present, in case of high-intensity use, Stress/Relaxation and Procrastination/Boredom were highly diminished, and Guilt/Shame was the dominant representation. Although the current version of the International Classification of Diseases (ICD-11) primarily focus on the behavioral and physical symptoms of substance use—such as impaired control, social impairment, and pharmacological criteria—negative emotional states that can be associated with substance use disorders are also included [69], thus guilt and shame may be considered as part of it. Similarly, guilt and shame are not explicitly mentioned in the I-PACE model, some of the elements of the model may indirectly address these emotions. For example, the cognitive component of the model includes beliefs and expectations related to substance use which may include negative self-evaluations and self-blame [70], which can lead to feelings of guilt and shame.

More frequent associations from the Guilt/Shame/Relief MR in case of high severity substance use is consistent with cyclical models of addiction that expect more problematic forms of substance use to be associated with higher levels of shame [77]. A possible explanation for the observed asymmetry between psychoactive substances and potentially addictive behaviors regarding guilt/shame as a function of problem intensity might be that guilt is an essentially social phenomenon [78], therefore it is probably more influenced by the highly stigmatized state of substance use (e.g., see [31]). It is important to note that the Stress/Relaxation MR decreased for both high-intensity behaviors and substance use, consistent with the predictions of Incentive Sensitization Theory (IST, [79]) that the proportion of positive feelings decreases with the development of addiction (for various problematic behaviors, such as gaming, excessive shopping and social media use [80]). Relaxation was the least frequent MR (see Table 4), with no difference between psychoactive substances and potentially addictive behaviors. The fact that only one–the rarest–MR reflected positive aspects is also in line with the assumptions of IST that addiction is usually not driven by positive emotions (e.g., [79]).

**Table 4:** Participants’ characteristics according to mental representation membership.

	Guilt/Shame/Relief	Addiction/Health	Stress/Relaxation	Procrastination/Boredom	*p*
n	229	138	74	180	
Age, mean ±SD	35 ± 10.7	37.1 ± 12.0	34.9 ± 10.5	33.4 ± 12.0	-
Women (%)	68.9%	71.0%	71.6%	73.8%	-
High intensity (%)	66.3%	74.2%	60.0%	60.4%	-
Substance (%)	37.7%	36.6%	15.0%	10.5%	< .001
Behavior (%)	35.3%	16.7%	10.2%	37.7%	
Most frequently mentioned associations					
1^st^	Guilt (88)	Addiction (64)	Calm (53)	Time wasting (123)	
2^nd^	Shame (55)	Pain (61)	Recreation (37)	Unnecessary (76)	
3^rd^	Weakness (31)	Harmful (60)	Joy (33)	Boredom (76)	
4^th^	Anxiety (30)	Expensive (54)	Good (26)	Constraints (43)	
5^th^	Frustration (29)	Unhealthy (47)	Stress (25)	Fatigue (42)	

The present study provides a comprehensive structure of *“disturbing excessive activity”-*related expressions, improving the understanding of individuals’ representations and emotions. The results indicated that a different focus of intervention might be required based on problem behavior type (substance use/non-substance-related potentially addictive behaviors) and the intensity of the problem. In the case of substance-related behaviors, a shift is present in the MRs from the physical (Addiction/Health) to the psychological (Guilt/Shame/Relief) harms. Since shame [81] and stigma [82] have been identified as a major factor for relapse and the failure to seek help [83], the results of the present study support that shame management is an important therapeutic step (e.g., [77,84,85]), especially in case of intensive substance users. Also, the associations and linked emotions of the Addiction/Health representation raises the possibility that health anxiety might be a comorbid condition of problematic use and addiction. This would not only contribute to personal suffering [86], but individuals may be at an increased risk of engaging in substance use when experiencing obsessive health concerns [87]. Consequently, its relationship to addiction should be further investigated. On the other hand, for high-intensity behaviors, Procrastination/Boredom was the most frequent MR. Therefore, interventions to improve time management skills might be a promising in reducing high-intensity engagement in different potential behavioral addictions.

Wang [53] reported that free-time management skills were protective against leisure boredom, which significantly predicted problematic internet use. Boredom has been reported to be a significant predictor of other problematic behaviors, such as pornography use [56], smartphone use [88], and internet gaming [89]. Also, Altiner et al. [90] reported a negative relationship between time management skills and the level of problematic internet use. At first glance, it may seem counter-intuitive that the Addiction/Health representation was less frequent for high-intensity compared to low intensity behaviors. This is partly explained by the fact that the frequency of Procrastination/Boredomrepresentation was higher, so the relative frequency of Addiction/Health necessarily decreased (i.e., an intense problematic behavior is more likely to trigger the concept of procrastination and boredomrelative to addiction and health). Another contributing factor might be that since quantitative norms for behaviors are harder to establish for individual or social levels than for psychoactive substance use, perceptions of being addicted to non-substance behaviors may be more driven by personal attitudes [91], making moral incongruence a relevant factor in the evaluation of potentially addictive behaviors (e.g., [92,93]).

A number of limitations to the present study warrant consideration. The data were all self-report and the sample was self-selected, both of which may have introduced methods biases (e.g., over-reporting or under-reporting, volunteer effects), potentially contributing to the high proportion of female participants. Despite the preprocessing efforts, which involved merging and translating the FWAs by two separate coders, the translations do not adequately account for the cultural disparities between the Hungarian and English languages. As a result, there may be minor inaccuracies in the outcomes. However, the primary objective of this analysis was not centered around individual FWAs, but rather on interpreting the collective FWAs within the modules, with the intention of minimizing inconsequential variations in translation. The present study did not test MRs and emotions explicitly across individuals with a clinical diagnosis of substance use disorder or behavioral addiction. Rather, potentially problematic use was classified as low-intensity or high-intensity based on a single item from the Screener for Substance and Behavioral Addictions [33]. Also, the data are not suitable to test more detailed associations between perceived wasted time and addiction-related measures (e.g., craving, motivation to quit, or subjective harm). Therefore, further studies are necessary to corroborate the assumption regarding perceived wasted time as a possible criterion when assessing for behavioral addictions.

Future studies should also consider the application of a longitudinal design, assessing use/behavior-related associations across multiple time-points. As the results of the present study suggest, changes in mental representations are to be expected, and exploring their temporal dynamics would be useful to better understand the emergence and evolution of maladaptive (addictive) behaviors. In addition, examining the temporal dynamics of mental representations may highlight important differences and similarities between the development of substance addictions and behavioral addictions, which may contribute to the theoretical basis for effective health communication and interventions. Further, to elaborate on the identified mental representations, qualitative research methods such as interviews and focus groups would be beneficial in gaining a more detailed understanding.

## Conclusion

The free word association approach combined with a data-driven co-occurrence network analysis is a promising technique in mapping MRs and feelings toward potentially addictive substances and behaviors. The distribution of Addiction/Health and Procrastination/Boredom mental representations varied significantly between substance use and other addictive behaviors, suggesting that different cognitive evaluation processes underlie them. The Addiction/Health representation was found to be more prevalent for substances, while the Procrastination/Boredom representation was more frequent for other addictive behaviors, and its frequency increased with the self-reported intensity of the behavior. Guilt/Shame/Relief was equally common for both substances and behaviors, but its likelihood increased with the intensity of substance use. These differences in the mental representations of substances and behaviors may have implications for the development of more accurate diagnostic components for potential behavioral addictions and could help address ongoing debates in the scientific literature, as noted by Billieux et al. (2015b).The common overlaps of representations for both psychoactive substances and potentially addictive behaviors further provides support for the scientific viewpoint that addiction may develop and be present even in the absence of psychoactive substances.

## Supporting information

S1 Appendix (XLSX)

S2 Appendix (DOCX)

## References

[1] Grant J. E., Potenza M. N., Weinstein A., & Gorelick D. A. (2010). Introduction to behavioral addictions. American Journal of Drug and Alcohol Abuse, 36(5), 233–241. doi: 10.3109/00952990.2010.491884 20560821 PMC3164585

[2] Brand M., Rumpf H. J., Demetrovics Z., MÜller A., Stark R., King D. L., et al. (2022). Which conditions should be considered as disorders in the International Classification of Diseases (ICD-11) designation of “other specified disorders due to addictive behaviors”? Journal of Behavioral Addictions, 11(2):150–159. 10.1556/2006.2020.00035 PMC9295220 32634114

[3] Gola M., Lewczuk K., Potenza M. N., Kingston D. A., Grubbs J. B., Stark R., et al. (2022). What should be included in the criteria for compulsive sexual behavior disorder? Journal of Behavioral Addictions, 11(2), 160–165. 10.1556/2006.2020.00090 PMC9295236 34329192

[4] Sassover E., & Weinstein A. (2022). Should compulsive sexual behavior (CSB) be considered as a behavioral addiction? A debate paper presenting the opposing view. Journal of Behavioral Addictions, 11(2), 166–179. 10.1556/2006.2020.00055 PMC9295215 32997646

[5] Stein D. J., & Lochner C. (2022). Nosology of behavioral addictions: Intersections with philosophy of psychiatry: Commentary to the debate: “Behavioral addictions in the ICD-11”. Journal of Behavioral Addictions, 11(2), 186–190. 35895455 10.1556/2006.2022.00033 PMC9295244

[6] Alavi S. S., Ferdosi M., Jannatifard F., Eslami M., Alaghemandan H., & Setare M. (2012). Behavioral addiction versus substance addiction: Correspondence of psychiatric and psychological views. International Journal of Preventive Medicine, 3(4), 290–294. 22624087 PMC3354400

[7] Starcevic V. (2016). Tolerance and withdrawal symptoms may not be helpful to enhance understanding of behavioural addictions. Addiction, 111(7), 1307–1308. doi: 10.1111/add.13381 27095413

[8] Billieux J., Maurage P., Lopez-Fernandez O., Kuss D. J., & Griffiths M. D. (2015a). Can disordered mobile phone use be considered a behavioral addiction? An update on current evidence and a comprehensive model for future research. Current Addiction Reports, 2(2), 156–162.

[9] Billieux J., Schimmenti A., Khazaal Y., Maurage P., & Heeren A. (2015b). Are we overpathologizing everyday life? A tenable blueprint for behavioral addiction research. Journal of Behavioral Addictions, 4(3), 119–123. doi: 10.1556/2006.4.2015.009 26014667 PMC4627665

[10] Griffiths M. D. (2019). The evolution of the ’components model of addiction’ and the need for a confirmatory approach in conceptualizing behavioral addictions. Düşünen Adam: The Journal of Psychiatry and Neurological Sciences, 32, 179–184.

[11] Kardefelt‐Winther D., Heeren A., Schimmenti A., van Rooij A., Maurage P., Carras M., et al. (2017). How can we conceptualize behavioural addiction without pathologizing common behaviours? Addiction, 112(10), 1709–1715. doi: 10.1111/add.13763 28198052 PMC5557689

[12] Griffiths M. D. (2010). The role of context in online gaming excess and addiction: Some case study evidence. International Journal of Mental Health and Addiction, 8, 119–125.

[13] Bőthe B., Lonza A., Štulhofer A., & Demetrovics Z. (2020). Symptoms of problematic pornography use in a sample of treatment considering and treatment non-considering men: A network approach. Journal of Sexual Medicine, 17(10), 2016–2028. doi: 10.1016/j.jsxm.2020.05.030 32675049

[14] Van Rooij A., & Prause N. (2014). A critical review of “Internet addiction” criteria with suggestions for the future. Journal of Behavioral Addictions, 3(4), 203–213. doi: 10.1556/JBA.3.2014.4.1 25592305 PMC4291825

[15] Petry N. M., Zajac K., & Ginley M. K. (2018). Behavioral addictions as mental disorders: To be or not to be? Annual Review of Clinical Psychology, 14, 399–423. doi: 10.1146/annurev-clinpsy-032816-045120 29734827 PMC5992581

[16] Baburajan V., e Silva J. D., Pereira F. C. (2020). Open-ended versus closed-ended responses: A comparison study using topic modeling and factor analysis. IEEE Transactions on Intelligent Transportation Systems, 22(4), 2123–2132.

[17] Dyevre A. (2021). Text-mining for lawyers: How machine learning techniques can advance our understanding of legal discourse. Erasmus Law Review, Forthcoming 14, 7.

[18] Grimmer J., & Stewart B. M. (2013). Text as data: The promise and pitfalls of automatic content analysis methods for political texts. Political Analysis, 21(3), 267–297.

[19] Stanga J. E., & Sheffield J. F. (1987). The myth of zero partisanship: Attitudes toward American political parties, 1964–84. American Journal of Political Science, 31(4), 829–855.

[20] Ames S. L. & Stacy A. W. (1998) Implicit cognition in the prediction of substance use among drug offenders. Psychology of Addictive Behaviors, 12, 272–281.

[21] Szalay L., Bovasso G., Vilov S., & Williams R. E. (1992). Assessing treatment effects through changes in perceptions and cognitive organization. American Journal of Drug and Alcohol Abuse, 18(4), 407–428. doi: 10.3109/00952999209051039 1449123

[22] Szalay L. B., Carroll J. F., & Tims F. (1993). Rediscovering free associations for use in psychotherapy. Psychotherapy: Theory, Research, Practice, Training, 30(2), 344.

[23] Stacy A. W., Leigh B. C., & Weingardt K. R. (1994). Memory accessibility and association of alcohol use and its positive outcomes. Experimental and Clinical Psychopharmacology, 2, 269–282.

[24] Reich R. R., Goldman M. S., & Noll J. A. (2004). Using the false memory paradigm to test two key elements of alcohol expectancy theory. Experimental and Clinical Psychopharmacology, 12(2), 102–110. doi: 10.1037/1064-1297.12.2.102 15122954

[25] Leung K. S., & McCusker C. G. (1999). Accessibility and availability of smoking-related associations in smokers. Addiction Research, 7(3), 213–226.

[26] Szalay L. B., & Deese J. (1978). Subjective meaning and culture: An assessment through word associations. Hillsdale: Erlbaum.

[27] Popoola I. O., Anders S., Feuereisen M. M., Savarese M., & Wismer W. V. (2021). Free word association perceptions of red meats; beef is ‘yummy’, bison is ‘lean game meat’, horse is ‘off limits’. Food Research International, 148, 110608. 34507752 10.1016/j.foodres.2021.110608

[28] File B., Keczer Z., Vancsó A., Bőthe B., Tóth-Király I., Hunyadi M., et al. (2019). Emergence of polarized opinions from free association networks. Behavior Research Methods, 51(1), 280–294. doi: 10.3758/s13428-018-1090-z 30094726 PMC6420605

[29] Gero D., File B., Hinrichs N., Mueller M., Ulbert I., Somogyvári Z., et al. (2020). Mental and emotional representations of “weight loss”: Free-word association networks in members of bariatric surgery-related social media communities. Surgery for Obesity and Related Diseases, 16(9), 1312–1320. doi: 10.1016/j.soard.2020.05.032 32665114

[30] Keczer Z., File B., Orosz G., & Zimbardo P. G. (2016). Social representations of hero and everyday hero: A network study from representative samples. PloS one, 11(8), e0159354. Advance online publication, doi: 10.1371/journal.pone.0159354 27525418 PMC4985139

[31] Yang L., Wong L. Y., Grivel M. M., & Hasin D. S. (2017). Stigma and substance use disorders: an international phenomenon. Current Opinion in Psychiatry, 30(5), 378–388 doi: 10.1097/YCO.0000000000000351 28700360 PMC5854406

[32] Thompson E. R. (2007). Development and validation of an internationally reliable short-form of the Positive and Negative Affect Schedule (PANAS). Journal of Cross-Cultural Psychology, 38(2), 227–242.

[33] Schluter M.G., Hodgins D.C., Wolfe J., Wild T.C. (2018). Can one simple questionnaire assess substance-related and behavioural addiction problems? Results of a proposed new screener for community epidemiology. Addiction, 113(8), 1528–1537. doi: 10.1111/add.14166 29357188

[34] Sarrica M. (2007). War and peace as social representations: Cues of structural stability. Peace and Conflict, 13, 251–272.

[35] Abric J. C. (1993). Central system, peripheral system: Their functions and roles in the dynamics of social representations. Papers on Social Representations, 2, 75–78.

[36] Blondel V. D., Guillaume J.-L., Lambiotte R., & Lefebvre E. (2008). Fast unfolding of communities in large networks. Journal of Statistical Mechanics, 2008, P10008, doi: 10.1088/1742-5468/2008/10/P10008

[37] Lancichinetti A., & Fortunato S. (2012). Consensus clustering in complex networks. Scientific Reports, 2(1), 1–7. doi: 10.1038/srep00336 22468223 PMC3313482

[38] The MathWorks Inc. (2022). MATLAB version: 9.13.0 (R2022b), Natick, Massachusetts: The MathWorks Inc. https://www.mathworks.com.

[39] Rubinov M., & Sporns O. (2010). Complex network measures of brain connectivity: uses and interpretations. Neuroimage, 52(3), 1059–1069. doi: 10.1016/j.neuroimage.2009.10.003 19819337

[40] Bastian M., Heymann S., & Jacomy M. (2009, March). Gephi: an open source software for exploring and manipulating networks. In Proceedings of the international AAAI conference on web and social media (Vol. 3, No. 1, pp. 361–362.

[41] Dearing R. L., Stuewig J., & Tangney J. P. (2005). On the importance of distinguishing shame from guilt: Relations to problematic alcohol and drug use. Addictive Behaviors, 30(7), 1392–1404. doi: 10.1016/j.addbeh.2005.02.002 16022935 PMC3106346

[42] Olivares‐Faúndez V. E., Gil‐Monte P. R., & Figueiredo‐Ferraz H. (2014). The mediating role of feelings of guilt in the relationship between burnout and the consumption of tobacco and alcohol. Japanese Psychological Research, 56(4), 340–348.

[43] Vaghefi I., Qahri-Saremi H., & Turel O. (2020). Dealing with social networking site addiction: a cognitive-affective model of discontinuance decisions. Internet Research. 30(5), 1427–1453.

[44] Sassover E., Abrahamovitch Z., Amsel Y., Halle D., Mishan Y., Efrati Y., et al. (2021). A study on the relationship between shame, guilt, self-criticism and compulsive sexual behaviour disorder. Current Psychology. Advance online publication, 10.1007/s12144-021-02188-3.

[45] Reid R. C., Stein J. A., & Carpenter B. N. (2011). Understanding the roles of shame and neuroticism in a patient sample of hypersexual men. Journal of Nervous and Mental Disease, 199(4), 263–267. doi: 10.1097/NMD.0b013e3182125b96 21451351

[46] Reid R. C., Temko J., Moghaddam J. F., & Fong T. W. (2014). Shame, rumination, and self-compassion in men assessed for hypersexual disorder. Journal of Psychiatric Practice, 20(4), 260–268. doi: 10.1097/01.pra.0000452562.98286.c5 25036581

[47] Gilliland R., South M., Carpenter B. N., & Hardy S. A. (2011). The roles of shame and guilt in hypersexual behavior. Sexual Addiction & Compulsivity, 18(1), 12–29.

[48] Dhuffar M. K., & Griffiths M. D. (2014). Understanding the role of shame and its consequences in female hypersexual behaviours: A pilot study. Journal of Behavioral Addictions, 3(4), 231–237. doi: 10.1556/JBA.3.2014.4.4 25592308 PMC4291828

[49] Tangney J. P., Stuewig J., & Mashek D. J. (2007). Moral emotions and moral behavior. Annual Review of Psychology, 58, 345–372. doi: 10.1146/annurev.psych.56.091103.070145 16953797 PMC3083636

[50] Maritz G. S., & Mutemwa M. (2012). Tobacco smoking: patterns, health consequences for adults, and the long-term health of the offspring. Global Journal of Health Science, 4(4), 62. doi: 10.5539/gjhs.v4n4p62 22980343 PMC4776909

[51] Patterson I., & Pegg S. (1999). Nothing to do: the relationship between’leisure boredom’and alcohol and drug addiction: is there a link to youth suicide in rural Australia? Youth Studies Australia, 18(2), 24–29.

[52] Lin C. H., Lin S. L., & Wu C. P. (2009). The effects of parental monitoring and leisure boredom on adolescents’internet addiction. Adolescence, 44(176), 993–1004. 20432612

[53] Wang W. C. (2019). Exploring the relationship among free-time management, leisure boredom, and internet addiction in undergraduates in Taiwan. Psychological Reports, 122(5), 1651–1665. doi: 10.1177/0033294118789034 30071775

[54] Chaney M. P., & Blalock A. C. (2006). Boredom proneness, social connectedness, and sexual addiction among men who have sex with male internet users. Journal of Addictions & Offender Counseling, 26(2), 111–122.

[55] Grubbs J. B., Wright P. J., Braden A. L., Wilt J. A., & Kraus S. W. (2019). Internet pornography use and sexual motivation: A systematic review and integration. Annals of the International Communication Association, 43(2), 117–155.

[56] Bőthe B., Tóth-Király I., Bella N., Potenza M. N., Demetrovics Z., & Orosz G. (2021). Why do people watch pornography? The motivational basis of pornography use. Psychology of Addictive Behaviors, 35(2), 172–186. doi: 10.1037/adb0000603 32730047

[57] Bocazi I. (2020). The effect of boredom proneness on smartphone addiction and impulse purchasing: A field study with young consumers in Turkey. Journal of Asian Finance, Economics, and Business, 7(7), 509–517.

[58] Brown S. A., & Munson E. (1987). Extroversion, anxiety and the perceived effects of alcohol. Journal of Studies on Alcohol, 48(3), 272–276. doi: 10.15288/jsa.1987.48.272 3657171

[59] McKennell A. C. (1970). Smoking motivation factors. British Journal of Social and Clinical Psychology, 9(1), 8–22. 5488969 10.1111/j.2044-8260.1970.tb00632.x

[60] Green B. O. B., Kavanagh D., & Young R. (2003). Being stoned: a review of self‐reported cannabis effects. Drug and Alcohol Review, 22(4), 453–460. doi: 10.1080/09595230310001613976 14660135

[61] Sinha R., & Jastreboff A. M. (2013). Stress as a common risk factor for obesity and addiction. Biological Psychiatry, 73(9), 827–835. doi: 10.1016/j.biopsych.2013.01.032 23541000 PMC3658316

[62] Demetrovics Z., Urbán R., Nagygyörgy K., Farkas J., Zilahy D., Mervó B., et al. (2011). Why do you play? The development of the motives for online gaming questionnaire (MOGQ). Behavior Research Methods, 43(3), 814–825. doi: 10.3758/s13428-011-0091-y 21487899

[63] Park J., Song Y., & Teng C. I. (2011). Exploring the links between personality traits and motivations to play online games. Cyberpsychology, Behavior, and Social Networking, 14(12), 747–751. doi: 10.1089/cyber.2010.0502 21780935

[64] Ozen H., & Engizek N. (2014). Shopping online without thinking: Being emotional or rational? Asia Pacific Journal of Marketing and Logistics, 26(1), 78–93.

[65] Sun S., Rubin A. M., & Haridakis P. M. (2008). The role of motivation and media involvement in explaining internet dependency. Journal of Broadcasting & Electronic Media, 52(3), 408–431.

[66] Whiting A., & Williams D. (2013). Why people use social media: a uses and gratifications approach. Qualitative Market Research, 16(4), 362–369.

[67] Ryding F. C., & Kaye L. K. (2018). “Internet addiction”: A conceptual minefield. International Journal of Mental Health and Addiction, 16(1), 225–232. doi: 10.1007/s11469-017-9811-6 29491771 PMC5814538

[68] Bőthe B., Tóth-Király I., Potenza M. N., Orosz G., & Demetrovics Z. (2020). High-frequency pornography use may not always be problematic. Journal of Sexual Medicine, 17(4), 793–811. doi: 10.1016/j.jsxm.2020.01.007 32033863

[69] World Health Organization (2019). International statistical classification of diseases and related health problems (11th revision). https://icd.who.int/en. 10.2471/BLT.25.294650 PMC13531092 42683092

[70] Brand M., Young K. S., Laier C., Wölfling K., & Potenza M. N. (2016). Integrating psychological and neurobiological considerations regarding the development and maintenance of specific Internet-use disorders: An Interaction of Person-Affect-Cognition-Execution (I-PACE) model. Neuroscience & Biobehavioral Reviews, 71, 252–266. doi: 10.1016/j.neubiorev.2016.08.033 27590829

[71] Griffiths M. D., & Duff J. S., (1993). Etiologies of excessive behaviour: A study of non-professional peoples’ beliefs. Addiction Research, 1(3), 199–206.

[72] American Psychiatric Association (2013). Diagnostic and statistical manual of mental disorders (5th ed.). American Psychiatric Publishing.

[73] Cargiulo T. (2007). Understanding the health impact of alcohol dependence. American Journal of Health-System Pharmacy, 64(5_Supplement_3), S5–S11. doi: 10.2146/ajhp060647 17322182

[74] Milcarz M., Polanska K., Bak-Romaniszyn L., & Kaleta D. (2018). Tobacco health risk awareness among socially disadvantaged people—A crucial tool for smoking cessation. International Journal of Environmental Research and Public Health, 15(10), 2244. doi: 10.3390/ijerph15102244 30322112 PMC6211097

[75] Rolova G., Gavurova B., & Petruzelka B. (2021). Health literacy, self-perceived health, and substance use behavior among young people with alcohol and substance use disorders. International Journal of Environmental Research and Public Health, 18(8), 4337. doi: 10.3390/ijerph18084337 33921885 PMC8073264

[76] Li W., O’Brien J. E., Snyder S. M., & Howard M. O. (2015). Characteristics of internet addiction/pathological internet use in US university students: a qualitative-method investigation. PloS One, 10(2), e0117372. 25647224 10.1371/journal.pone.0117372 PMC4315426

[77] Luoma J. B., Chwyl C., & Kaplan J. (2019). Substance use and shame: A systematic and meta-analytic review. Clinical Psychology Review, 70, 1–12. doi: 10.1016/j.cpr.2019.03.002 30856404

[78] Baumeister R. F., Stillwell A. M., & Heatherton T. F. (1994). Guilt: An interpersonal approach. Psychological Bulletin, 115(2), 243–267. doi: 10.1037/0033-2909.115.2.243 8165271

[79] Robinson T. E., & Berridge K. C. (1993). The neural basis of drug craving: an incentive-sensitization theory of addiction. Brain Research Reviews, 18(3), 247–291. doi: 10.1016/0165-0173(93)90013-p 8401595

[80] File D., Bőthe B., File B. & Demetrovics Z. (2022). The role of impulsivity and reward deficiency in “liking” and “wanting” of potentially problematic behaviors and substance uses. Frontiers in Psychiatry, 13, 820836. doi: 10.3389/fpsyt.2022.820836 35546934 PMC9083266

[81] Brown B. S. (1991). Relapse prevention in substance misuse: Introduction. International Journal of the Addictions, 25(9A-10A), 1081–1083. 10.3109/10826089109081038 1966680

[82] Chang K. C., Chang Y. H., Yen C. F., Chen J. S., Chen P. J., Lin C. Y., et al. (2022). A longitudinal study of the effects of problematic smartphone use on social functioning among people with schizophrenia: Mediating roles for sleep quality and self-stigma. Journal of Behavioral Addictions, 11(2), 567–576. doi: 10.1556/2006.2022.00012 35394922 PMC9295235

[83] Potter-Efron R., & Potter-Efron P. (2013). The treatment of shame and guilt in alcoholism counseling. Routledge.

[84] Wilson M. (2000). Creativity and shame reduction in sex addiction treatment. Sexual Addiction & Compulsivity, 7(4), 229–248.

[85] Adams K. M., & Robinson D. W. (2001). Shame reduction, affect regulation, and sexual boundary development: Essential building blocks of sexual addiction treatment. Sexual Addiction & Compulsivity, 8(1), 23–44.

[86] Taylor S., Asmundson G. J., & Hyprochondria. (2004). Treating health anxiety: A cognitive-behavioral approach New York: Guilford Press.

[87] Palmer G. A., & Daiss D. D. (2005). Personality characteristics of adolescents with hallucinogen, methamphetamine, and cannabis dependence: A comparative study. Journal of Child & Adolescent Substance Abuse, 15(1), 37–49.

[88] Casale S., Fioravanti G., & Spada M. M. (2021). Modelling the contribution of metacognitions and expectancies to problematic smartphone use. Journal of Behavioral Addictions, 10(3), 788–798. doi: 10.1556/2006.2021.00066 34613932 PMC8997219

[89] Li L., Niu Z., Griffiths M. D., Wang W., Chang C., & Mei S. (2021). A network perspective on the relationship between gaming disorder, depression, alexithymia, boredom, and loneliness among a sample of Chinese university students. Technology in Society, 67, 101740.

[90] Altiner Yas M., Isci N., Alacam B., Caliskan R., & Kulekci E. (2021). Relationship between level of internet addiction and time management skills among nursing students. Perspectives in Psychiatric Care, 58(2), 758–766. doi: 10.1111/ppc.12845 33974273

[91] Vaillancourt-Morel M. P., & Bergeron S. (2019). Self-perceived problematic pornography use: Beyond individual differences and religiosity. Archives of Sexual Behavior, 48(2), 437–441. doi: 10.1007/s10508-018-1292-6 30116928

[92] Grubbs J. B., & Perry S. L. (2019). Moral incongruence and pornography use: A critical review and integration. Journal of Sex Research, 56(1), 29–37. doi: 10.1080/00224499.2018.1427204 29412013

[93] Lewczuk K., Nowakowska I., Lewandowska K., Potenza M. N., & Gola M. (2021). Frequency of use, moral incongruence and religiosity and their relationships with self‐perceived addiction to pornography, internet use, social networking and online gaming. Addiction, 116(4), 889–899. doi: 10.1111/add.15272 32955140

