# Supplementary material for: Investigating mental representations of psychoactive substance use and other potentially addictive behaviors using a data driven network-based clustering method
Source: PLoS One. 2023 Oct 19;18(10):e0287564. doi: 10.1371/journal.pone.0287564 (PMC10586681; doi:10.1371/journal.pone.0287564)
Supplement: S2 Appendix — (DOCX) [file pone.0287564.s002.docx]

Appendix 1.


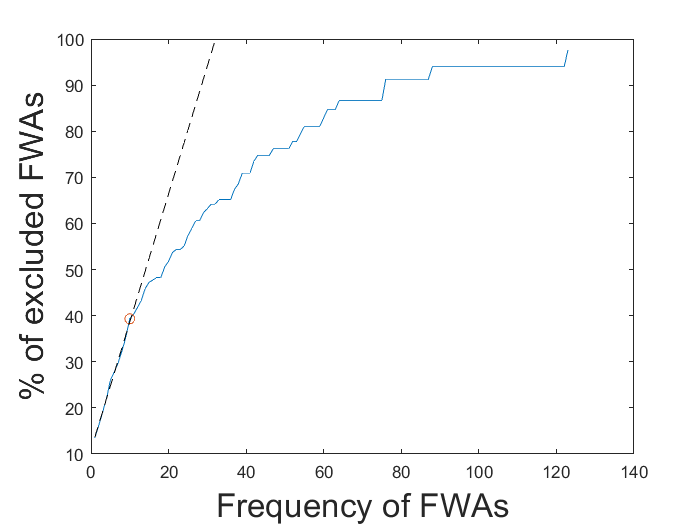


Figure Appendix. Relationship between the frequency of FWAs and the percentage of excluded FWAs. The data exhibits a breakpoint at a frequency of 10, as indicated by a linear fit to the function.

Appendix 2.

Kruskal-Wallis test was used to compare modules across the 14 emotions which covered together 49% of the PANAS labels. To control for multiple comparisons, Bonferroni corrected p values of 0.003 were used (0.05/14). The distribution of the following emotions were different across MRs: ‘mad’ [H=17.6, df=3, p<0.001]; ‘frightened’ [H=21.6, df=3, p<0.001]; ‘apathy’ [H=34.1, df=3, p<0.001]; ‘excited’ [H=19.41, df=3, p<0.001]; ‘grateful’ [H=31.82, df=3, p<0.001];

The distribution of the following emotions were not different across MRs: ‘upset’ [H=9.23, df=3, p=0.026]; ‘proud’ [H=2.15, df=3, p=0.54]; ‘generous’ [H=1.87, df=3, p=0.59]; ‘hopeful’ [H=11.2, df=3, p=0.01]; interested [H=9.71, df=3, p=0.02]; ‘hostile’ [H=13.43, df=3, p=0.004]; ‘emphaty’ [H=3.48, df=3, p=0.32]; ‘envy’ [H=9.81, df=3, p=0.02]; ‘sympathy’ [H=5.61, df=3, p=0.13].

For Pairwise comparisons see Table Appendix.
